# An Update on Recent Geriatric Education Advocacy Efforts

**DOI:** 10.1093/geroni/igaa057.1802

**Published:** 2020-12-16

**Authors:** Catherine Carrico, Katherine Bennett

**Affiliations:** 1 University of Wyoming, Laramie, Wyoming, United States; 2 University of Washington, Seattle, Washington, United States

## Abstract

The National Association for Geriatric Education (NAGE) has maintained consistent education and advocacy efforts since 2006. In recent years NAGE has implemented formal and grassroots advocacy strategies. At the federal level NAGE has increased collaboration with other aging advocacy organizations and coalitions. At the request of Congress, NAGE leadership and stakeholders have testified before Congress and regularly submit testimony to the House and Senate. NAGE staff maintain strong working relationships with congressional staff. Strategies for effective grassroots education and advocacy have been taught to members, and membership has mobilized to educate elected officials about the essential work of the Geriatric Workforce Enhancement Programs across the country. This presentation will provide a thorough review of NAGE’s advocacy work over the past 4 years.

